# Characterization of Asia 1 sdAb from Camels Bactrianus (*C. bactrianus*) and Conjugation with Quantum Dots for Imaging FMDV in BHK-21 Cells

**DOI:** 10.1371/journal.pone.0063500

**Published:** 2013-05-30

**Authors:** Shuanghui Yin, Shunli Yang, Youjun Shang, Shiqi Sun, Guangqing Zhou, Ye Jin, Hong Tian, Jinyan Wu, Xiangtao Liu

**Affiliations:** State Key Laboratory of Veterinary Etiological Biology, National Foot and Mouth Diseases Reference Laboratory, Lanzhou Veterinary Research Institute, Chinese Academy of Agricultural Sciences, Lanzhou, Gansu, China; University of North Carolina Greensboro, United States of America

## Abstract

Foot-and-mouth disease (FMD), caused by FMD virus (FMDV), is a highly contagious viral disease affecting cloven-hoofed animals. Camelids have a unique immunoglobulin profile, with the smallest functional heavy-chain antibodies (sdAb or VHH) naturally devoid of light chains with antigen-binding capacity. We screened and characterized five sdAbs against FMDV by immunized library from *C. bactrianus* with Asia 1 virus-like particles (VLPs). Three of five recombinant sdAbs were stably expressed in *E.coli*, remained highly soluble, and were serotype-specific for VP1 protein of FMDV Asia 1 by ELISA. These failed to completely neutralize the Asia 1 virus. According to the *K_D_* value of binding affinity to three sdAbs, which ranged from 0.44 to 0.71 nm by SPR, sdAb-C6 was selected and conjugated with Zn/CdSe quantum dots (QDs) to form a QDs-C6 probe, which was used to trace and image the subcellular location of FMDV in BHK-21 cells. The results show that FMD virions were observed from 3 h.p.i., and most of virions were distributed on one side of the nucleus in the cytoplasm. We demonstrate the utility of sdAbs as functionalized QDs are powerful tools for FMDV research.

## Introduction

Foot-and-mouth disease (FMD) is a highly contagious viral disease of cloven-hoofed livestock, caused by FMD virus (FMDV), a member of the genus *Aphthovirus,* in the family *Picronaviridae*. It consists of a single-stranded, positive-sense RNA genome of approximately 8.5 kb surrounded by four structural proteins (VP1 to 4), which form viral capsid proteins that are considered antigenic, in addition to 15 different, mature non-structural proteins (Nsp) [1]. There are currently seven known serotypes: A, O, C, Asia 1, and three South African Territories (STA1, SAT2 and SAT3), and multiple subtypes exist within each serotype [2], [3]. The recent outbreaks of FMD, especially in a number of FMD-free regions, such as Taiwan (1997) [4] and the United Kingdom (2001) [5], have caused huge economic losses and significantly increased public awareness. However, in FMD-free countries, livestock, including cloven-hooved animals, are extremely susceptible to FMDV due to the non-prophylactic-vaccination policy, which results in no humoral response against FMDV. Globally, FMDV serotypes O and the A are the most prevalent [6], [7]. However, Asia has its own unique serotype, and outbreaks due to Asia 1 have been reported only sporadically in the past few decades. Analysis of Asia 1 indicates that some strains have been spread across large distances between countries in Asia within a short time [8].

Camelids produce conventional antibodies, but they also contain a high quantity of naturally occurring, functional antibodies devoid of light chains [9]. The evolutionary advantage of such heavy chain antibodies (HcAb) is still unclear. In HcAb, the fragment antigen binding (Fab) region is reduced to a single variable domain (sdAb, VHH, or Nanobodies, Nbs) which is easily cloned and produced by standard genetic techniques [10]. The sdAb displays very significant characteristics in many selection systems such as phage, yeast, or ribosomal display [11]. Of particular note is the small size and strict monomeric behavior, and when combined with other biochemical properties such as high solubility while readily expressed in *Escherichia coli* (*E. coli*), a high specificity and affinity makes an advantageous tool for many medical and biotechnological applications, including proteomics [10].

Quantum dots (QDs) are semiconductor nanocrystals (2 to10 nm), such as CdSe/ZnS cores, which emit fluorescence depending on their particle size, provide brighter and more stable fluorophores that are comparable with fluorescent proteins [12], [13]. QDs are luminescent inorganic fluorophores which allow for multicolor imaging, but orthogonal targeting approaches must be developed to label different proteins with different color QDs. Antibody-conjugated-QDs represent one approach for protein labeling. However, compared to conjugating full-length monoclonal antibodies (mAb) and single chain antibody fragments (ScFv) with sdAbs, which is a relatively easy process because sdAbs are the smallest (≈15 kDa) known natural domain with full available antigen-binding capacity [14]. Due to their small size, sdAb molecules have been shown to act as strong enzyme inhibitors by interacting with enzyme pockets not accessible to common antibodies [15], and have the ability to efficiently target and aid in imaging of specific immune cell types *in vivo* [16]. Recombinant sdAb fragments are emerging as new tools for sensitive diagnostics methods, molecular probes, and infectious disease therapy.

Here, we describe the preparation, isolation, selection, and characterization of FMDV serotype Asia 1 by binding recombinant sdAbs derived from peripheral mononuclear lymphocytes (PMLC) of *Camelus bactrianus* (*C. bactrianus*) and generating five sdAbs with high affinity with FMDV antigen by phage display. These were expressed in *E. coli* and purified, and three of the five sdAbs (sdAb-C4,C5 and C6) expressed soluble protein. The identical results demonstrated that these are type-specific against FMDV Asia 1 by ELISA. Furthermore, we used CdSe/ZnS core-shell QDs with emission wavelengths of 605 nm (QD605, red) conjugated with the sdAb-C6, a highly sensitive, newly developed nanoprobe, to achieve target detection and fluorescent spectral analysis of FMDV infection in BHK-21 cells.

## Results

### Detection for camels FMDV Asia 1 antibody

Two Bactrian camels were appropriated from the Gobi desert before starting the experiment, and they nor their parents were vaccinated against FMDV, and were as such considered “natural specific pathogen free (nSPF)” animals. The camels were immunized with recombinant VLPs expressing antigenic epitopes of FMDV Asia 1. Blood samples were collected and serum was isolated from both camels at several time points throughout the study. The serum samples were tested by LPB-ELISA for time-sensitive evaluation of the animals' immune reaction against the antigen. The results indicate that a continuous and high immune reactivity was observed, and that seroconversion occurred after secondary inoculations. Interestingly, antibody titration of the female camel demonstrated a higher reactivity than the male (Table 1).

**Table 1:** Immunization and antibody response against FMDV type Asia1 in *C. Bactrianus*.

Time	Dose (μg/each)	Interval (days)	Antibody titre (♂/♀)
Primary	50	0^a^	1:16/1:16
1^st^ booster	80	21^b^	1:256/1:256
2^nd^ booster	110	35^c^	1:512/1:1024
3^rd^ booster	200	49^d^	1:1024/1:2048*

Note: 0^a^ day serum before the primary vaccination; 21^b^ days, 35^c^ days, 49^d^ days which were after the primary immunization, respectively; *Peripheral blood was collected 7 days after the 3^rd^ inoculation.

### Construction of VHH library

To construct a VHH library for FMDV Asia 1, the 6×10^8^ PBMC of the camels, underwent extraction of total RNA from which cDNA synthesized. The VH and V_H_H regions were amplified by three rounds PCR. The cDNA was a template in the first PCR with the first PCR primers, based on available protein sequence information and on the camel V_H_H clone VH-CH2, and was predicted to anneal to codons 1 through 10 of the camel V_H_H of subgroup III, the only family reported to be present in camels. The 600 bp fragment (VHH-CH2 without the CH1 exon) was purified from 1.8% agarose gel after separation from the 900 bp fragment (VH-CH1-CH2 exons). The VHH fragments in the 600 bp gene were a template for amplification by using the second PCR primers, which anneal at the framework 1 and framework 4 regions. The third PCR primers, which contain the *Sf*iI/*Not*I restriction sites and the 450 bp gene fragments were cloned into the phagemid vector pHEN2, and were transformed into *E. coli* TG1. A strong enriching effect was observed, and dilution plating of the cultured library indicated a total of 1.91×10^5^ colonies. By coating the intact virus directly, a polyclonal phage ELISA was conducted. Twenty-four clones were then picked up from the third elution and subjected to monoclonal phage ELISA to bind the intact virus positively and specifically. By local DNA sequencing, 5 unique sdAb genes were identified (GenBank: sdAb-C3/C4/C5/C6/C20, KC816013/ KC816014/ KC816015/ KC816016/ KC816017).

**Figure 1:**
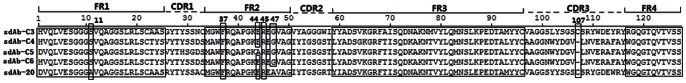
Multiple amino acid sequence alignment of FMDV Asia 1 specific sdAbs antibody clones. Amino acid sequences were aligned according to the Kabat numbering. The specific VHH amino acids were thick-lined boxed, the FRs were thin-lined boxed.

In this work, we first screened five specific sdAb antibodies against FMDV Asia 1 from camels by phage display. Analysis of sdAb amino acid fragments showed that the four conservative hallmark residues of sdAb in FR2 were Phe37, Glu44Gly/Ala, Arg45 and Ala47Gly. With the exception of three samples, residues in FR2 of one sequence were mutated into a Ala in sdAb-C5 and sdAb-20, and these substitutions of otherwise conserved amino acids are hallmarks of sdAb originating from heavy-chain homodimer immunoglobulins [17]. Furthermore, the CDR3 consisted of lengths of 19 and 20 amino acids, and two of the five clones contain a Cys107 (sdAb-C3 and 20) which could possibly form a disulfide bond with a second cysteine located in the CDR1(Fig. 1). In contrast to human VHs, the camelid VHHs are generally well expressed in *E. coli*, highly soluble, and stable at high concentrations in aqueous solutions. Comparison of the crystal structures of cAbAn33 and its humanized derivative reveals steric hindrance exerted by VHH-specific residues Tyr37 and Arg45, which prevent the VL domain pairing, whereas Glu44 and Arg45 are key elements to avoid insolubility of the domain [18]. In our study, although sdAb-C3 and 20 have distinguished features of sdAb-derived from camels and also were expressed in *E. coli*, the former is a soluble protein that could not bind the resin and the latter is insoluble.

### Expression and purification soluble sdAb antibody proteins

**Figure 2:**
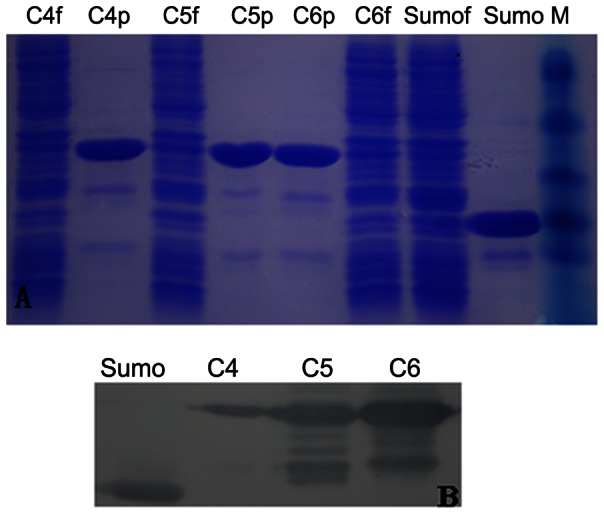
Analysis of purified the three sdAb proteins by SDS-PAGE. Lane sdAb-4f, 5f, 6f and Sumof, flow though; Lane sdAb-4p, 5p, 6p and Sumop, purified the single-domain antibody proteins with Ni-NTA agarose; M, protein molecular weight standards, the size markers are 85, 50, 35, 25 and 20 kDa from top to bottom (A). Western-blot analysis of products of recombinant fusion single-domain antibody protein (B).

The three unique sdAb fragments were cloned into vector p-SMK, and transformed into BL21-codon-Plus (DE3)-RIL strains, which were induced with IPTG for 20 h at 20°C. Solubility of the recombinant sdAb protein were indentified by SDS-PAGE, and confirmed its apparent molecular weight and that the recombinant proteins of clone 4/5/6 express solubility behavior (Fig. 2A). Unfortunately, clone 3/20 both were inclusion bodies (data not shown). The purified recombinant proteins of clone 4/5/6 were stained using NTA affinity resins which have the ability to specifically bind to His_6_ tag polypeptides (Fig. 2A). The fusion tag of all three clones (Clone 4/5/6) may be recognized with an anti-His antibody and observed by Western blotting (Fig. 2B).

### Identification of Asia 1 sdAb

The dasELISA assay and guinea pig-antisera as a parallel positive control was able to demonstrate the ability of sdAb to bind FMDV Asia 1 antigen, as indicated by sdAb having similar characteristics with polyclonal anti-sera, and both were able to capture target antigen. There was no significant difference in reading, suggesting that the sdAb is effective when used as a capture antibody.

**Figure 3:**
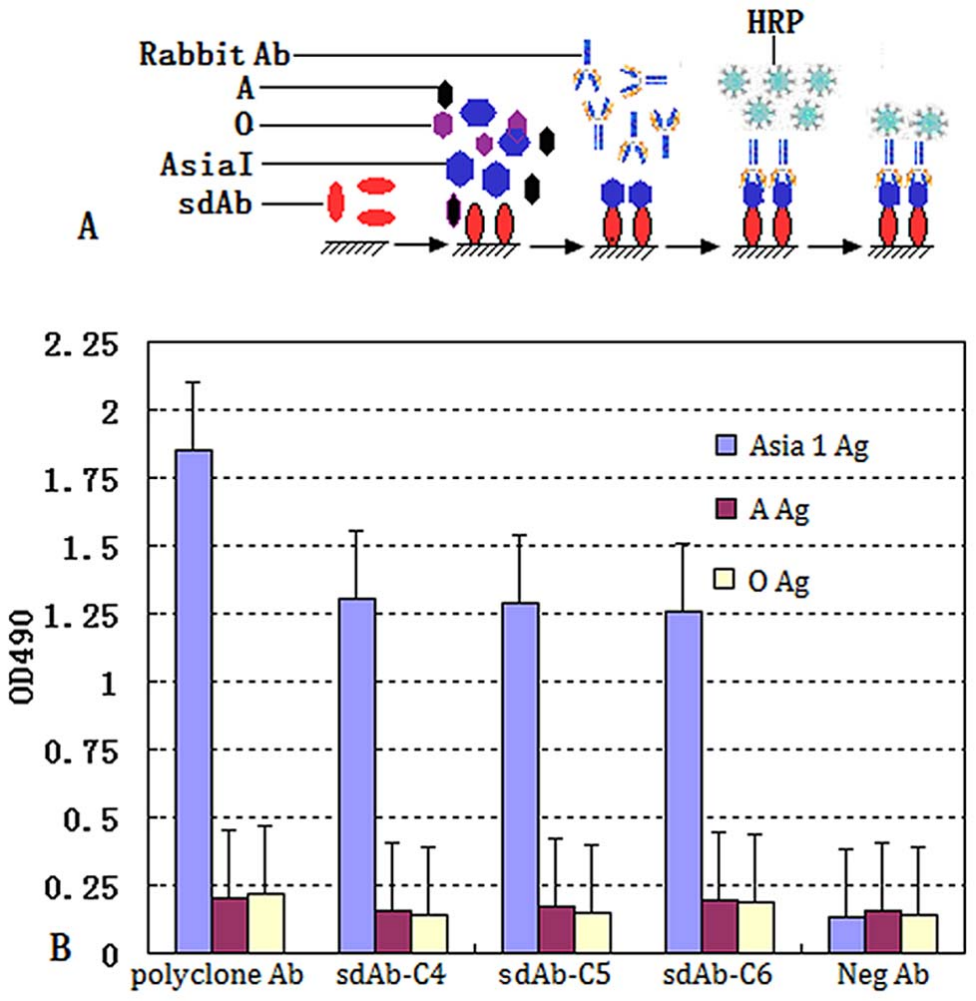
The dasELISA was carried out for an identification of sdAb serotype-specific against FMDV Asia 1 with A, and O. Schematic overview of strategy to identify serotype specific Asia 1 of FMDV using dasELISA (A). Polyclonal Ab of Asia 1 coated as positive control, the well of Neg Ab from camels' serum coated as negative control, and the wells of sdAb-C4/C5/C6 as capture Ab for binding the Asia 1 antigen. All samples were tested for absorbance at 450 nm (B).

The same dasELISA method was used to study the serotype-specific capability of sdAb with FMDV serotypes A, O, and Asia 1 inactive antigens. Our results indicate that sdAb has high specificity against Asia 1 and exhibits no cross-reactivity with serotypes O and A antigen (Fig. 3).

VNT results demonstrated that all sdAb failed to neutralize the FMDV Asia 1, and sdAb was only able to delay cytopathic effect within the cell cultures for 3–4 h when compared to the positive control. The reactivity of the sdAbs to recombinant FMDV-Asia 1-VP1 and VP0-3 was examined using an indirect ELISA. Our results indicate that three sdAbs bound only to recombinant Asia 1-VP1 only (Table 2).

**Table 2:** VNT and kinetic of sdAbs interaction with FMDV Asia 1 antigen.

sd Ab	aVNT	VP1	VP0- VP3	*K*a× 10^−5^	*K*d× 10^−4^	*K*_D_	^b^EL ISA
		OD_450_	OD_450_	[M^−1^S^−1^]	[S^−1^]	[nM]	OD_450_
sdAb-C4	NO	1.37	0.23	9.29	4.32	0.47	1.702
sdAb -C5	NO	1.55	0.21	7.37	5.21	0.71	1.48
sdAb -C6	NO	1.98	0.174	16	7.03	0.44	1.891

a VNT: All sdAb fragments failed to neutralize the FMDV, and were only able to delay cytopathic effect to 3–4 h p.i. when compared to the control. ^b^ ELISA: the OD_450_ values were obtained from sdAb screening of all clones binding to inactivated Asia 1 antigen by

The affinities of sdAb ranged from 0.44 to 0.71 nM (Table 2), which is within the range of *K_D_* values most sdAbs have for their targeted antigens. The results demonstrate the *K_D_* value of binding affinity of sdAbs to native antigen, which is consistent with previous screening sdAbs by ^b^ELISA (Table 2). Therefore, these data on identify clone 6 as an optimal candidate for further antibody research.

### Characteristics of CdSe/ZnS ODs conjugated anti-Asia1 sdAb

**Figure 4:**
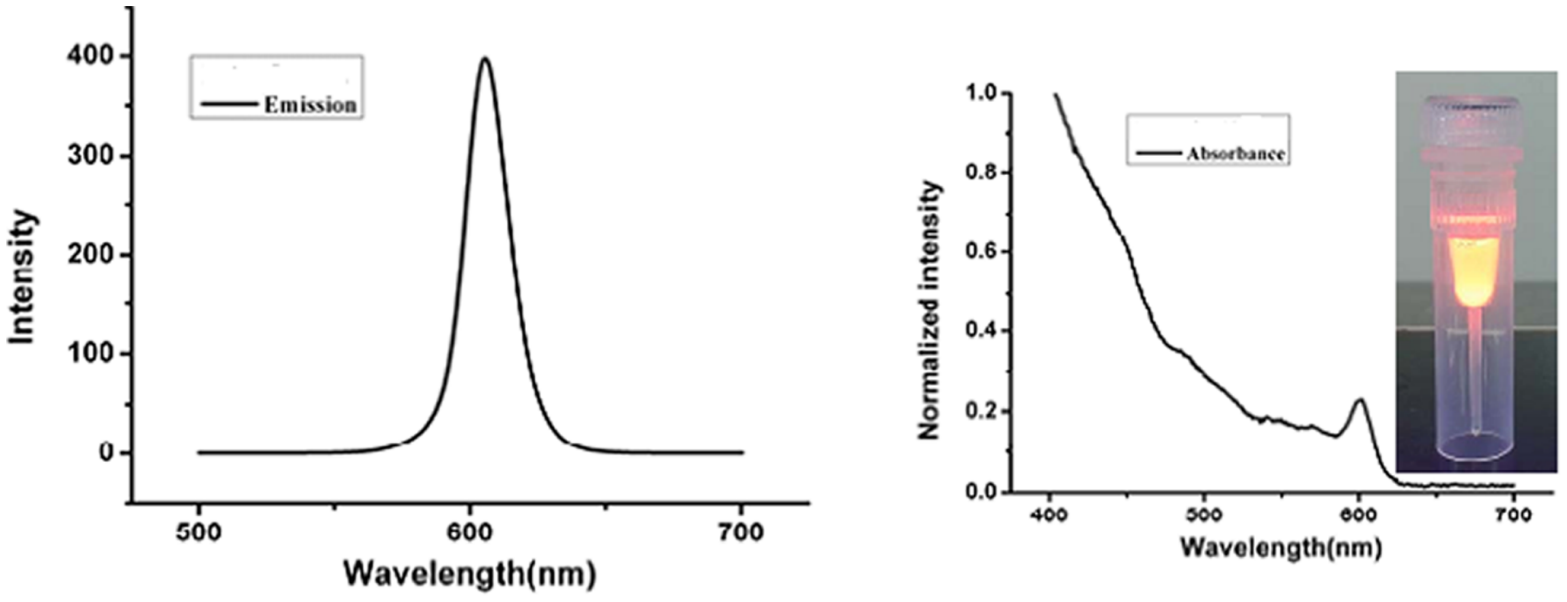
Emission spectra of quantum dots labeled sdAb-C6 (A). (84329A:Em = 605 nm,fwhm = 19 nm;84329B) UV-vis absorption spectra of QDs labeled sdAb-C6 (B).

Absorption spectra were acquired on a Perkin Elmer LS-55 UV/vis spectrometer run on the Olympus BX51, DP72. (Fig. 4), and demonstrates the optical properties including absorption and emission of the CdSe/ZnS QDs conjugated with anti-Asia 1 sdAb dispersed in 50mM borate at pH 8.4. We also tested whether these sdAbs are labeled successfully with QDs by 1% agarose gel and these coatings are run relatively slowly because of bigger molecular weight than QDs alone (data no show), indicating sdAbs conjugation with QDs.

### ODs-sdAb probe imaging FMDV Asia1 in BHK-21 cells

To establish the kinetics of FMDV infection in BHK-21 cells and study the interactive characteristics of QDs-sdAb probe, FMDV-infected BHK-21 cells were monitored for the presence of virions over time by immunofluorescence (IF) microscopy.

**Figure 5:**
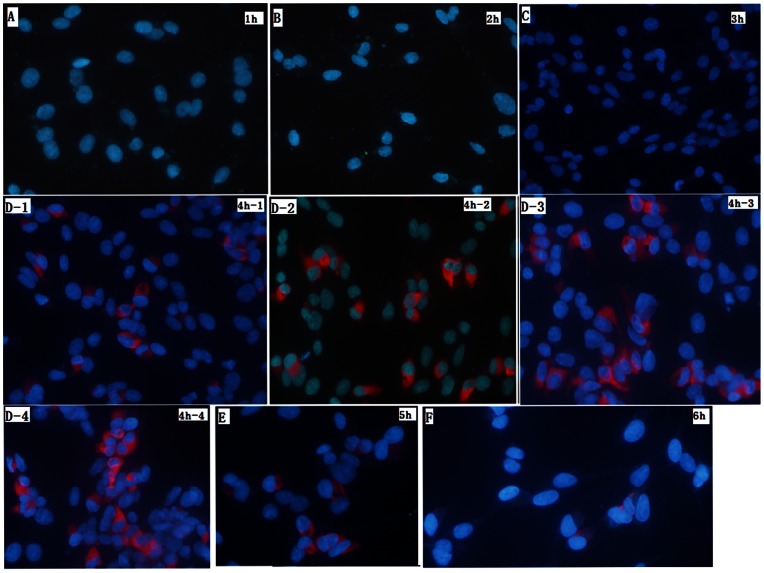
Images of distribution of FMDV type Asia1 in BHK-21 cells using the QDs-C6 probe. Monolayers of BHK-21 cells were infected with FMDV type Asia 1 for 1 h at 37°C and processed for double IF staining as described in Material and Methods. FMDV virions were localized with specific visualized ODs-C6 probe against the capside VP1 (red), the nucleus was stained with DPI. Specific VP1 immunostaining was not detected form 1 h to 2 h p.i. (A-1 h and B-2 h) to VP1 A small number of FMDV virion could be observed from 3 h p.i. (C-3 h), at 4 h p.i, at the stage of infection the major of cells were infected by FMDV, and virions accumulated on one side of the nucleus (D-4 h-1,-2,-3 and –4). At the late phage of infection, small remanants of infected cells were stained (E-5 h and F-6 h). Zoom is ×40.

The first and second images indicate that the FMD virions cannot be observed with QD-sdAb staining (Fig. 5) at 1 and 2 h p.i., although we inspected for virions in all fields on all coverslips. With lastingness of infection, the minority of cells in cultures had developed virion at 3 h p.i. the image in Fig. 5 shows that FMDV virions appeared sporadically in perinuclear sites (red).

At 4 h p.i., the majority of BHK-21 cells demonstrated detectable FMD viral capsid protein labeling with QD-sdAb probe indicating that the viral protein isolated to a specific region on one side of the nucleus. As the time reached 5 h p.i. and 6 h p.i., the majority of cells, both infected and non-infected, had detached from the coverslips, and consequently the detectable number of cells became less visible.

**Figure 6:**
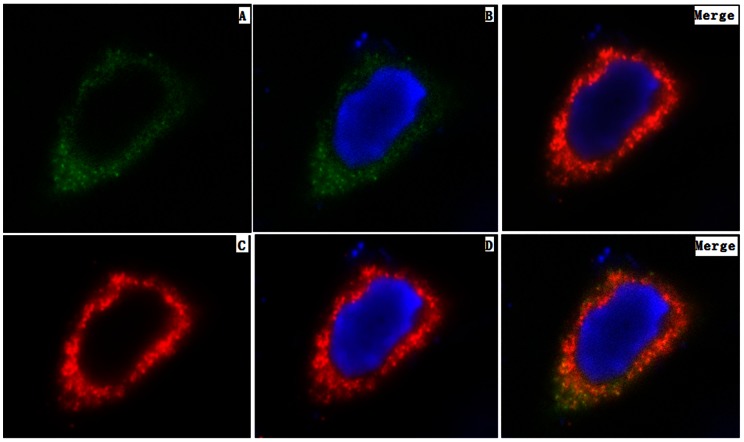
Subcellular localization of FMDV using Alexa Fluor-488-conjugated-sdAb-C6 and QDs-C6 probe. BHK-21 cells infection with FMDV Asia1 at 4 h p.i. were fixed and stained for double-label immunofluorescence using the AF488-C6 (green, A and B) and QDs-C6 (red, C and D) probe specific for VP1. FMDV virions were visualized by double staining at the same region. Panles showed merged images. Zoom is×100.

To identify the QD-sdAb probe *in vitro*, a standard fluorescence detection probe, AF488-C6, was conjugated with sdAb-C6, and dubbed AF488-C6 probe (Fig. 6). This was used to demonstrate that the FMD viral proteins were visible, through the green AF488-C6 probe in BHK-21 cells at 4 h p.i. The green AF488-C6 probe was able to stain the FMD virions in the same location with QD-sdAb probe in cells. This demonstrated that the QD-sdAb nanoprobe specifically bound FMD Asia 1 virions. In addition, IF studies identified strong staining with QD-sdAb probe for newly synthesized capsid proteins of FMDV, and provided clear discrimination between the virions and nucleus.

## Discussion

The Office International des Épizooties (OIE) Code chapter on FMD includes camelids as being susceptible species to FMD, giving the impression that they are similar to cloven-hoofed animals in their potential involvement in the epidemiology of FMD [19]. In fact, dromedary camels are not susceptible to FMDV type A infection and to neither be a reservoir of FMDV nor transmit this virus to susceptible species. When compared with the non-susceptible dromedaries, Bactrian camels showed moderate to severe clinical signs of FMD and developed high titers of antibodies to FMDV 7–10 days post-inoculation [20]. Days post-inoculation [20]. *C bactrianus* are more likely to induce strong passive immune reactions against FMDV antigen than other camels in our experience. *C bactrianus* produce high titers of antibodies post-immunization with Asia 1 antigen rendering them the ideal candidate animal for the production of sdAb against FMDV.

In this report, we demonstrate that mature virions can be reliably recognized and labeled with QD-sdAb probe in BHK-21 cells infected with FMDV Asia 1 up to 3 h.p.i. There is a dramatic distribution of the cytoplasmic contents, leading to a very defined location pattern that has been associated with the viral replication complex. For many positive-strand viruses, a common feature of infection is the extensive rearrangement of host cell membranes, and generation of cytoplasmic vesicles which are apparently required for replication. Most research performed on picornaviruses is based on analysis of cells infected with poliovirus [21]. FMDV, as member of the *Picornaviridae*, has a similar genome organization and is believed to follow a similar replication strategy to poliovirus [22]. In contrast to poliovirus, replication and translation of FMDV RNA occurs in the cytoplasm [23], and a replication complex of FMDV and its possible association with cellular membranes has not yet been described in detail. However, our research with a QD-sdAb probe demonstrates that mature FMD virions are located on one side of the cell nucleus as the time after inoculation increases. This has been similarly reported for serotype O FMDV, and since the site is often occupied by the Golgi apparatus where it is described as the virus replication site, it has become the primary focal point of the subsequent events that take place within the FMDV-infected cell [24]. Differences in the kinetics of expression and cell distribution among FMDV Nsp have been observed in BHK-21-infected cells. 3D^pol^ was the first protein detected at 1.5 h p.i., and it appeared in a perinuclear distribution. Between 2 to 2.5 h p.i., 2B, 2C, 3B, and 3C were detected, mostly exhibiting a punctated and scattered pattern, while 3A and 3D^pol^ showed concentration to one side of the nucleus [25].

FMDV mRNA is translated by a cap-independent mechanism that utilizes an internal ribosomal entry site, and taking over the host cell protein synthesis machinery [26] which antagonizes the cellular innate immune and inflammatory responses to the viral infection and produces virus progeny [27]. The leader proteinase (L^pro^) of FMDV is involved in overcoming the innate immune response by blocking the expression of interferon (IFN) and disturbing IFN-β transcription during FMDV infection. Interestingly, FMDV L^pro^ is localized to similar nuclear regions globally, and the transcription factors are situated in close proximity during viral infection [28]. BHK-21 or IBRS-2 cell lines are typically used for the propagation of FMDV. Analyses of both cell lines indicates that they harbor either an impairment in type IIFN production or IFN response, and do not allow a clear phenotypic differentiation between wild-type virus and leaderless viruses [29]. Therefore, FMDV, as with other RNA viruses, dependents on various host cell factors for their replication, and as modify a variety of cellular signal transduction pathways. To counter some of the host immune responses, viruses have developed very sophisticated mechanisms to subvert the host defense and to support their own replication [30].

The sdAb fragment is a VH variable domain that is approximately one-tenth the size of the conventionally assumed humoral respondent IgG (15 kDa vs 150 kDa) [31], and are called nanobodies due to their lack of the Fc fragment which renders them unable to exhibit nonspecific binding to target antigen. This feature is likely causing the weak virus neutralizing activity of the anti-FMDV sdAbs *in vitro*. However, blocking of viral cell entry, which is the predominant mechanism of *in vitro* FMDV neutralization, requires far higher antibody concentration than are normally reached *in vivo* [32]. While sdAb are capable of binding their antigens with affinities comparable to conventional IgG, its size allows it to bind epitopes inaccessible to conventional IgG. sdAbs are characterized by a low tendency to aggregate [33], exist as monomers, disseminate much better in tissues than full-size IgG [34], increase the ability withstand harsh physiochemical treatments, maintains solubility under certain non-physiological conditions, and resume normal function following exposure to chemical heat or heat denaturing [35], [36]. All these advantages make sdAb the best capture molecules due to the great deal of flexibility in their chemistry for bioconjugation to QD-based fluorescent nanoprobes for biodetection and diagnostics. Zamman et al (2009) developed sdAb-conjugated QDs as nanoprobes for cellular imaging of cancers cells, which bind strongly to epidermal growth factor (EGF) receptor, a protein which is widely accepted as a tumor marker [37]. QD-sdAb probes have a highly oriented manner, which make them an attractive alternative for the generation of ultra-small targeted nanoprobes in high-throughput diagnostic platforms [38].

sdAb is as a new class of molecular tracers, which are routinely indentified with nanomolar affinity for their target, and are easily tailored for molecular imaging and drug delivery application. Molecular imaging is aimed at the noninvasive investigation of cellular and molecular events in living subjects. The key advantage of this imaging methodology is that a biological process can be investigated in its native environment in an intact living individual (animal or human) [39]. Rothbauer et al (2006) prepared a GFP sdAb from alpacas (*Lama pacos*) as a probe of chromobodies for targeting and tracing antigens in different subcellular compartments in living cells, which can be targeted and traced for any potentially antigenic cellular structure [40]. Additionally, sdAbs can successfully target brain epitopes by transmigrating through blood-brain barrier. [41], can be used for tuning and detecting the activity of cell proteins in vivo [42], [43], [44], provide better diffusion in fixed cells in comparison to conventional antibodies [34], and can simplify the generation of anti-idiotypic antibodies suitable for vaccination [45].

Finally, the latest antibody engineering designs for sdAb, such as diabodies, minibodies, and labeling tags, have achieved impressive stability and specifity, and are now perfectly positioned to take full advantage of the next-generation position-labeling chemistries and QD conjugations to open new opportunities in a variety of research and amplification fields.

## Conclusions

We have defined a panel of specific sdAb (Nanobody) for FMDV serotype Asia 1 from C. bactrianus by Western blotting, ELISA, VNT and SPR. The sdAb-C6 was selected as a candidate antibody to conjugate with QDs forming QDs-sdAb probe, which was used to locate and image the subcellular distribution of FMDV Aisa1 in BHK-21 cells. The results demonstrate clearly visible FMD virions from 3 h.p.i., onward and most virions were distributed to one side of the nucleus in the cytoplasm. Herein, we establish the utility of sdAbs as functionalized QDs are powerful tools for FMDV research and others pathogens. The confluence of nanotechnology and biomaterials, which helps researchers understand the many basic biological processes and phenomena in level of visible by nano luminescent materials to label nanobody, then conjugate products to bind potential targets. Therefore, the new biomaterial probe is helpful example for strengthening the sdAbs applications for tracing the virus or cancer cells or others, and our research also expand the range of nanobody as in vivo tools for virus infection and functional advantages of sdAb.

### Ethics Statement

All animals were handled in strict accordance with good animal practice according to the Animal Ethics Procedures and Guidelines of the People's Republic of China, and the study was approved by the Animal Ethics Committee of Lanzhou Veterinary Research Institute, Chinese Academy of Agricultural Sciences (No. LVRIAEC2012-006). In this study, camles were permitted as experimental animal by the owner.

## Materials and Methods

### Viral antigen

All of the FMDV strains, sera, and ELISA assay used in this study are reference strains obtained from OIE/CHINA National Foot and Mouth Disease Reference Laboratory.

The virus-like particles (VLPs) from Asia 1 (Strain:1/Jiangsu/China/2005; Genbank: EF149009) were used as antigen in immune animals, and were developed from a recombinant expression of specific structural proteins (VP1 and VP0-VP3) in an *E. coli* expression vector.

### *C. bactrianus* immunization and antibody detection

Bactrian camel feeding, management, immunization, and sample collections were conducted by trained personnel under the supervision of a veterinarian.

Two Bactrian camels (one year old male and ten month old female) were immunized with Asia 1 VLP antigen preparations, emulsified in a 206 adjuvant. Camels were inoculated intramuscularly in the back, both sides of the neck, and on both hind legs at different sites on days 0, 21, 28, 35 and 42. Serum samples from peripheral blood via jugular venipuncture were collected on day 0 and subsequently after each inoculation. The sera antibody response was monitored by FMDV type Asia 1 liquid-phase blocking (LPB) ELISA.

### PMLC preparation

A total of 600 ml of peripheral blood was collected from the external jugular vein in EDTA coated tubes, 7 days after the final inoculation (300 ml from each camel and pooled). Total PBLCs were isolated by Ficoll-Paque gradient centrifugation; cell pellets were suspended in MEM medium, then transferred into 6-cell plates and incubated for 30 min at 37°C to eliminate cell debris. Monoclonal cells were divided into aliquots of 6×10^8^ PBLC, and were subsequently stored in liquid nitrogen.

### RNA extraction, PCR amplification and sdAb antibody Library Construction

The total RNA was extracted by using a commercially availabe RNA extraction kit (Nucleospin RNA II; Macherey Nagel). First-strand cDNA was synthesized by using Superscript III reverse transcriptase with oligo(dT)_12–18_ primers (Invitrogen). The cDNA encoding sdAb and VH was specifically amplified with the first PCR primers H1 and G1 (Table 3), annealing at both the leader and CH2 sequences. The 600 bp gene encoding for sdAb fragments was the template for amplification by using the second PCR primers, H2 and H3, which anneal at the framework 1 and 4 regions, and were followed by the third PCR primers, P1 and TN, which contain the restriction sites for further cloning steps. The final PCR fragments contained the *Sfi*I/*Not*I restriction sites, and were ligated into the phage vector pHEN2. Ligated material was transformed in triplicate into *E. coli* TG1 cells. The colonies from the plated cells were collected, washed, and stored at –80°C in LB medium, supplemented with glycerol equivalent to 50% of the final concentration.

**Table 3:** Primers used for amplification of sdAbs.

Primer	Primer sequence (5′–3′)	Gene (bp)
H1	gtcctggctgctcttctacaagg	
G1	ggtacgtgctgttgaactgttcc	900/600
H2	ggattgttattactcgcggcccagccggccatggctSAKgtgcagctggtggagtctgg	
H3	gccccgtgatggtgatgatgatgtgcggctgaggagacRgtgaccWG	450
P1	ggattgttattactcgcg	
TN	agcggccgcgacactggactacagatcctcttctgagatgagtttttgttctgcggccccg-tgatggtgatgatg	450

Specific sdAbs against FMDV type Asia 1 were selected from the immune sdAb library using phage display technology. The library was infected with M13K07 helper phages, and phage particles expressing the sdAb repertoire were rescued and precipitated with polyethylene glycol. Enrichment of specific binding points was performed by three rounds of *in vitro* selection. The VLPs antigen of serotype Asia 1 was coated directly and a pre-made phage display camel sdAb library was screened to find specific binding points. After three rounds of screening, a strong enriching effect was observed by quality control polyclonal phage ELISA. While screening for Asia 1 specific sdAb fragments, phages displaying the selected sdAb were selected and separated from monoclonal TG1 *E. coli* clones by phage ELISA. Unique sdAb were identified by local DNA sequencing.

### Expression and purification of recombinant sdAb

sdAb cDNA of the unique clones was amplified by PCR, and were sub-cloned into p-SMK vector with a fusion tag of small ubiquitin-like modifiers (Sumo) and expressed in *E. coli* [46]. Briefly, five pairs of expression primers of sdAb were designed for amplification of sdAb sequences (Table 4), which was purified and digested with restriction enzymes, cloned into p-SMK. The recombinant plasmids were transformed into BL21-codon-Plus (DE3)-RIL strain (Stratagene). The culture was induced by isopropyl-β-thiogalactopyranoside (IPTG) and incubated for 20 h at 20°C with constant agitation of 180 rpm. Cells were separated by centrifugation and the pellet was resuspended in 50 mM Tris-Cl buffer (pH 8.0). The cells were then cracked with an ultrasonic cell crusher, and the collected supernatant was purified using NTA affinity resins. The results were analyzed through 12% SDS-PAGE and Western blotting.

**Table 4:** Primers for expression sdAbs.

Primer	Sequence(5′-3′)	Gene (bp)	Enzyme site
sdAb-3	CTTGAAGACACAGGTCATGTGCAGCTGGTGGAG		*Bbs*I
	AGTGGATCCTCATGAGGAGACGGTGACCTG	381	*Bam*HI
sdAb -4	CTTCGTCTCAAGGTCATGTGCAGCTGGTGGAG		*Bsm*BI
	AGTGGATCCTCATGAGGAGACAGTGACCTGG	378	*Bam*HI
sdAb -5	CTTCGTCTCAAGGTGATGTGCAGCTGGTGGAG		*Bsm*BI
	AGTGGATCCTCATGAGGAGACAGTGACCTGG	378	*Bam*HI
sdAb -6	CTTCGTCTCAAGGTCATGTGCAGCTGGTGGAG		*Bsm*BI
	AGTGGATCCTCATGAGGAGACAGTGACCTGG	378	*Bam*HI
sdAb -20	CTTCGTCTCAAGGTGATGTGCAGCTGGTGGAG		*Bsm*BI
	AGTGGATCCTCATGAGGAGACAGTGACCTGG	381	*Bam*HI

### Western blotting

Three pairs of soluble recombinant sdAb antibody protein were transferred to an Immobilon-P Transfer membrane (Millipore Corporation, USA) in transfer buffer (20mM Tris-HCl, 190 mM glycine, 0.1% SDS, 20% Methanol, pH 8.3). The membrane was blocked for 1 h with PBST (containing 0.05% Tween 20, 5% skim milk) and incubated with 1∶1000 anti-His monoclonal antibody by a peroxidase-conjuated goat-anti-mouse antibody (antiHis-HRP, Sigma, USA) diluted by PBST. The assay was used to develop the signal by DAB reagent.

### Characterization of FMDV Asia1 sdAb

#### Identification of serotype-specific using double antibody sandwich ELISA

The double antibody sandwich ELISA (dasELISA) assay was carried out for identification of sdAb against specific serotypes, including FMDV Asia 1 as well as A and O. 96-well microtiter plates were coated with capture antibodies, including sdAb against FMDV Asia 1, rabbit-antisera of Asia 1, A, and O diluted at1:1000 for serotype Asia 1, A, and O; sdAbs of C4, C5, and C6 were diluted to 2.12 μg/μl, 3.75 μg/μl, and 0.84 μg/μl, respectively, in 0.06 M carbomate/bicarbonate buffer (pH 9.6). They were then incubated overnight at 4°C. Viral antigens of FMDV Asia 1 (inactivated FMDV), A, and O were added into wells. Three serotype-specific guinea pig antiseras of FMDV were added to the corresponding wells as positive controls, and two wells were selected to be coated with the capture antibodies and the Sumo recombinant protein (1∶2000, antiHis-HRP against Sumo His-tag) as negative controls. The corresponding the serotype-specific rabbit antisera against FMDV were added to all wells. The HRP conjugated goat anti-rabbit HRP IgG was added to all wells. After colorimetric reaction with TMB as substrate, the reaction was stopped with 2.0 M sulfuric acid, and the absorbance was measured at 450 nm.

#### Neutralization capacity of sdAb

A virus neutralization test (VNT) was performed as described previously [47].The LPB ELISA was carried out according to standard procedure for sdAbs as previously described [48]. The dilutions of antisera required to affect 50% inhibition were calculated for all the viruses [49].

#### Indirect ELISA for locating the sdAb origin

An indirect ELISA is the common method of detection, and was performed. FMDV Asia 1 recombinant antigens (VP1 at 6.47 mg/ml, VP0-VP3 7.85 mg/ml) were coated onto 96-well microtiter plates and were incubated overnight at 4°C. The plates were blocked with PBST buffer (5% skim milk, 10% *E. coli* lysate with 0.05% Tween 20) at room temperature for 1 h. The sdAbs were then added to the plates. After this incubation period, an antiHis-HRP mAb, at dilution of 1∶2000, was added followed by TMB substrate, and the reaction was stopped with 2.0M sulfuric acid. The optical absorbance was measured at 450 nm.

#### Affinity of the sdAb for FMDV

The affinity of the sdAb s selected to bind Asia 1 antigen was determined by surface plasmon resonance (SPR) on a Biacore 3000 (Biacore, Uppsala, Sweden). Asia 1 VLPs_antigen was directly immobilized to 1000 resonance units on the reactive surface of the CM5 sensor chip. The antibody fragments were diluted in HEPES-buffered saline at concentrations between 25 and 500 nM, and were subsequently injected at a flow rate of 30 μl/min over the sensor chip to record their binding kinetics toward the antigen [50]. The obtained data were analyzed with BIA evaluation software version 4.1. The kinetic rate constants (*Ka* and *Kd*), were recorded to calculate the equilibrium dissociation constant (*K_D_*).

### CdSe/ZnS ODs conjugated anti-Asia1 sdAb

ZnS-capped-CdSe ODs (QDs, 605 nm) were synthesized by Wuhan Jiayuan QuantumDots Co. LTD, Wuhan, China [51]. These activated QDs modified with thioglycolic acid were dissolved in 50 mM borate (8 μM, pH 8.4) containing 50mmol EDC (1-(3-dimethl-aminopropyl)-3-tehylcarbodiimide hydrochloride, >98%). The resulting QDs were used to observe the optimized wide ranging criteria. The QDs were reacted with 20 μl sdAb-C6, which were conjugated with solution of high-quality oil-soluble core-shell QD605 (QDs-C6), at room temperature (RT) in a shaking incubator for 2 to 4 h. The final QD bioconjugates were purified by centrifugation at 6000 g for 10 min, and the supernatant was transferred into an ultra-filtration tube (MW 30 kDa) to retain 50 kDa molecular and remove unreacted sdAb and impurities. The samples were then dialyzed at 4°C and underwent centrifugation at 2000 g until the appropriate volume was reached. The results were determined and visualized by using the optimized wide-ranging criteria and agarose gel. The QDs-C6 probes were stored at 4°C.

QDs-C6 based fluorescence probe detection was also compared to the standard fluorescence probe, Alexa Fluor-488-conjugated-sdAb-C6 (AF488-C6), to further determine the sdAb-C6 conjugated antibody specificity against FMDV in cells. The standard procedure was used to label the sdAb antibodies with AF488-C6 as previously described.

### QDs-C6 probes imaging FMDV Asia1 in BHK-21 cells

Monolayers of baby hamster kidney-21 (BHK-21) cells were seeded on coverslips in 6-well plates, and were infected with 10^6^ TCID_50_ Asia 1virus diluted in DMEM as previously described [52]. After 1 h of adsorption, the culture supernatant was removed and the monolayer was washed twice with fresh medium containing 0.5% fetal calf serum, and cells were incubated at 37°C with 5% CO_2_. Cell samples were harvested at 1 h, 2 h, 3 h, 4 h, 5 h, and 6 h post-infection (p.i.). All samples on coverslips were fixed with 4% paraformaldehyde, rinsed with 25 mM glycine (pH 7.4), and permeabilized with 0.1% Triton X-100, 1% bovine serum albumin, and 1M glycine in PBS for 15 min at RT. These were incubated in blocking buffer (5% normal goat serum, 2% bovine serum albumin, 10 mM glycine) to reduce unspecific binding for 2 h at RT.

The processed cells were then incubated with QDs-sdAb diluted in 1% BSA in PBS for 1 h in a humidified chamber at RT. After washing three times in PBS, nuclei were stained with 1 μg/ml of DAPI (4,6-diamidino-2-phenylindole, Invitrogen). Following immunochemical staining, the coverslips were mounted using the Prolong Antifade Kit (Molecular Probes), and observed under an Olympus BX51 fluorescence microscope coupled to a digital camera.
